# DESIGNING INTERGENERATIONAL LEARNING PLACES: STRATEGIES AND OUTCOMES AT AN AGE-FRIENDLY UNIVERSITY

**DOI:** 10.1093/geroni/igae098.0183

**Published:** 2024-12-31

**Authors:** Tamar Shovali

**Affiliations:** Eckerd College, St Petersburg, Florida, United States

## Abstract

The idea that universities play a vital role in promoting intergenerational learning and reciprocal sharing of knowledge among learners of all ages is a key principle of the Age Friendly University model. Although there is abundant research on the benefits of intergenerational relationships and learning in the classroom there is less information about details of how to transform a classroom space into a place where these relationships can occur. This suggests a need for reports of practical approaches in this area. I will describe an intergenerational team-based strategy employed with first year undergraduates in an intensive 3-week course. Students collaborated in teams across the semester to design an intergenerational activity tied to Age Friendly University principles to execute at the end of the semester. To assess the quality of intergenerational learning I surveyed students and older participants about the impact of the project on themselves, the College, and the local community. Prior to engaging in the projects the majority of students (66.7%) and older adults (66.7%) reported occasionally interacting with the other. Both reported an increase in the quality of their intergenerational interactions, students by 50% and older adults by 22.3%. Older and younger learners agreed that intergenerational projects benefit the College and the wider community beyond the College. Qualitative results about outcomes of this programming highlighted friendships and intergenerational communication and learning from younger learners and enjoyment and hope from older learners. Necessary to success in developing effective course-based programming are intentional design and ongoing evaluation.

